# Successful management of a giant retroperitoneal ancient schwannoma mimicking malignant tumors: A case report and literature review

**DOI:** 10.1097/MS9.0000000000001445

**Published:** 2023-11-01

**Authors:** Nooshin Zaresharifi, Sahand Karimzadhagh, Ramin Ebrahimian, Zoheir Reihanian, Elahe Abbaspour, Paridokht Karimian, Jouan Taheri Talesh

**Affiliations:** a Department of Pathology; b Department of Neurosurgery; c Department of Surgery, Faculty of Medicine; d Clinical Research Development Unit of Poursina Hospital, Guilan University of Medical Sciences; e Department of Pathology, Anatomical and Clinical Pathologist, Razi Laboratory, Rasht, Iran

**Keywords:** case report, giant schwannoma, neurilemmoma, retroperitoneal schwannoma

## Abstract

**Introduction and Importance:**

Schwannomas, originating from Schwann cells surrounding nerve sheaths, tend to be slow-growing. Among these, giant ancient schwannomas are remarkable for their rare occurrence and their capacity for substantial growth and regressive changes. Furthermore, the expansiveness and flexibility of the retroperitoneal space often conceal the symptoms of retroperitoneal schwannomas, leading to delayed diagnosis and allowing these tumors to grow significantly and become large and long-standing before detection.

**Case Presentation:**

A 24-year-old man presented with left flank pain and a growing abdominal bulge in the left upper quadrant. Computed tomography scan revealed a 15×15×10 cm lytic expansile lesion. Consequently, the encapsulated mass was surgically excised and diagnosed as an ancient retroperitoneal schwannoma through histological and immunohistochemical studies.

**Clinical Discussion:**

Comprehensive preoperative planning and a multidisciplinary strategy are imperative for the complete excision of schwannomas. These tumors can present diagnostic complexities, particularly due to nuclear atypia and pleomorphism, which might lead to misinterpretation regarding malignancy. Despite the risks associated with percutaneous biopsies, the low mitotic count is a critical diagnostic factor. Our study underscores the consensus that the definitive diagnosis should rely on postoperative histopathological findings, highlighting the importance of accurate assessment.

**Conclusion:**

Rare giant retroperitoneal ancient schwannomas pose diagnostic challenges due to their rarity, lack of distinct symptoms, and atypical locations. This study presents a successful case and management.

## Introduction

Highlights

- Retroperitoneal schwannomas are rarely encountered tumors, making up around 1–3% of the total cases of schwannomas.
- The diagnosis of rare giant retroperitoneal ancient schwannomas is complex due to their infrequency, absence of clear symptoms, and uncommon locations.
- The main approach involves a complete surgical excision to preserve neurological function and reduce postoperative complications.
- Nuclear changes and pleomorphism in these tumors can lead to the misdiagnosis of malignancy. The low mitotic count is vital for accurate assessment in such cases.
- A confirmed ancient retroperitoneal schwannoma, measuring 15×15×10 cm, was completely excised from a 24-year-old man.

Schwannomas, derived from Schwann cells within nerve sheaths, are tumors that grow slowly over time and typically do not exhibit any symptoms^1^. These tumors are predominantly benign in nature, but it is worth noting that malignant forms have been documented in up to 60% of individuals with Von Recklinghausen’s disease (Neurofibromatosis type 1)^2^. Giant solitary schwannomas are uncommon and can vary in size^3^. They are characterized by their involvement of two or more adjacent spinal levels or their size exceeding 2.5 cm in the largest dimension. Regarding clinical presentation, schwannomas display nonspecific symptoms, with patients experiencing localized pain and pressure-related symptoms in the advanced delayed tumor detection^4^.

Ancient schwannomas, a unique tumor subtype, are characterized by their remarkable growth and frequent regressive changes, including fatty degeneration, cyst development, and hemorrhage. These distinctive features differentiate them from other tumor types^5^.

Retroperitoneal schwannomas (RSs) are infrequent. They often go unnoticed due to the expansive and flexible nature of the retroperitoneal space, leading to delayed diagnosis and significant lesion growth^6^.

The primary treatment strategy involves surgical removal, which can be achieved through either traditional open procedures or minimally invasive laparoscopy, typically resulting in favorable therapeutic outcomes. This process requires comprehensive preoperative planning and a multidisciplinary approach due to the intricate nature of both diagnosis and treatment^7,8^. Due to their rare occurrence, lack of specific manifestations, and involvement of unusual sites, they pose a significant challenge for diagnosis. Therefore, this paper aims to report a rare giant retroperitoneal ancient schwannoma case and detail its successful management. This work has been reported in line with the Surgical CAse REport (SCARE) criteria^9^.

## Case presentation

A 24-year-old man was admitted to our hospital with a 1-year history of abdominal bulging in his left upper quadrant and a progressive enlargement over the previous several weeks. The patient reported the bulging as painless and denied other symptoms like nausea, vomiting, or changes in bowel habits, with the exception of occasional flank pain. He had no personal or family history of malignancy. The physical examination showed a pulse of 88 beats per minute, body temperature of 37.2°C, respiration of 20 breaths per minute, and a blood pressure of 110/90 mmHg.

**Figure 1:**
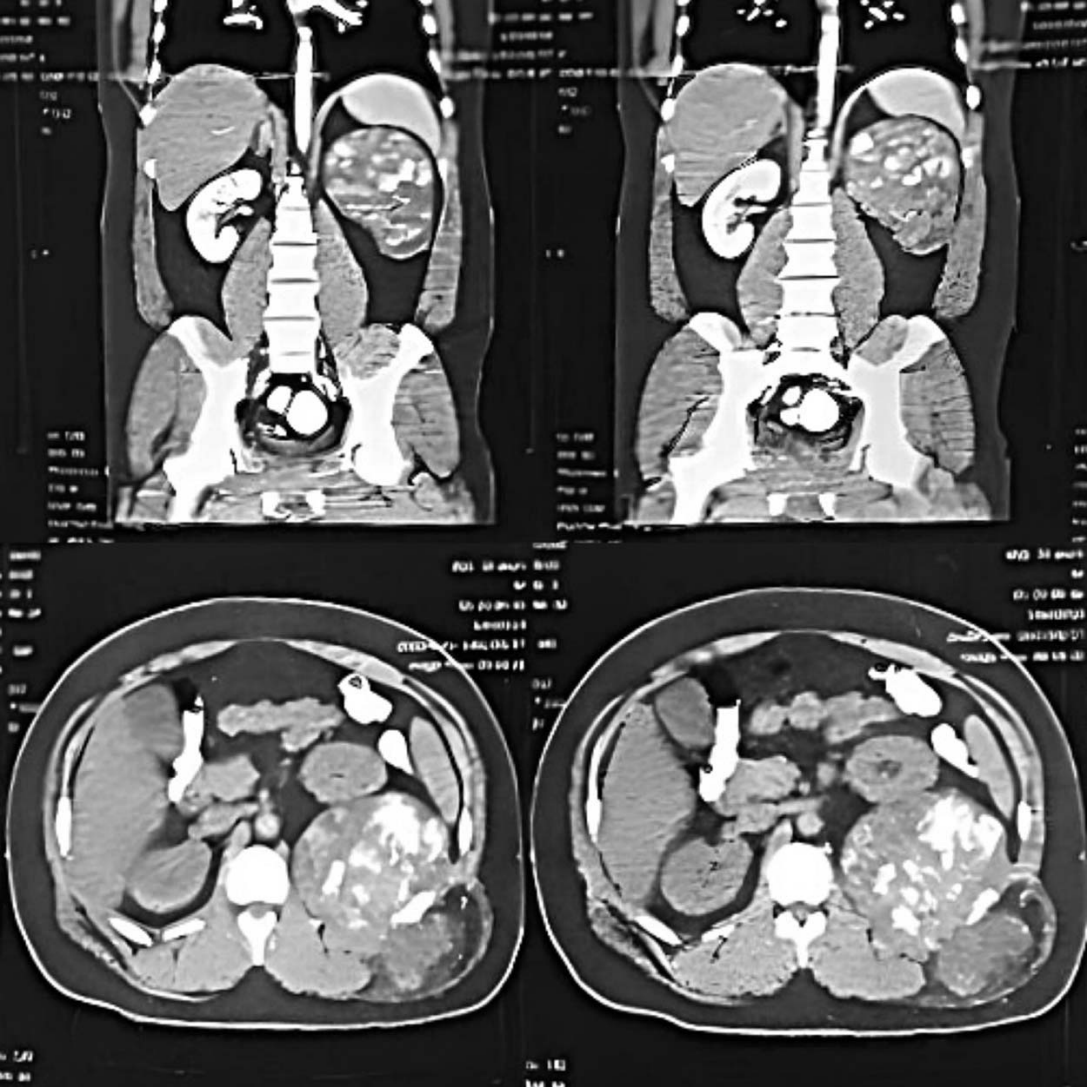
Preoperative computed tomography (CT) scan showed the presence of a lytic expansile lesion with multiple internal high densities from the left lower ribs.

Upon palpation, a substantial mass was detected below the patient’s left lower ribs. His laboratory tests were in the normal range except for a slight leukocytosis. Abdominal ultrasonography revealed an irregularly shaped echo, measuring 121×66 mm, located posterior to the left kidney. Color Doppler imaging displayed vascularity associated with the mass. A computed tomography (CT) scan identified a lytic expansile lesion in the vicinity of the left lower ribs, characterized by multiple internal high-density areas (Fig. 1).

These findings raised suspicion of bone-derived malignancies such as chondrosarcoma or intra-abdominal mesenchymal sarcoma, considering the mass effect on the left kidney and its anteromedial displacement. The patient subsequently underwent an ultrasound-guided core needle biopsy, which was consistent with a histopathologic diagnosis of Schwannoma tumor.

Eventually, the decision to complete surgical resection was made. Under general anesthesia, the patient was placed in a lateral position. An ~20 cm long incision was carefully made to access and decompress retroperitoneal structures and address the tumor’s attachment to the 12th rib with a thorough separation of the intercostal muscles, layer by layer.

In our case, the operation’s main goal was to expose the tumor clearly and to resect it entirely without injuring the left kidney. However, there were challenges in separating the tumor from the bottom of the posterior thoracic wall and the 12th rib. Due to the adhesion, the distal part of the 12th rib was also resected along with the tumor. Moreover, the diaphragm was partially deflected and required repairing by suturing. Accordingly, the encapsulated mass, measuring 15×15×10 cm in diameter, was excised entirely. To pre-empt potential postoperative complications like pneumothorax and hemothorax, a chest tube was inserted, and subsequently, the chest was closed. After the surgery, the patient experienced an uneventful recovery.

**Figure 2:**
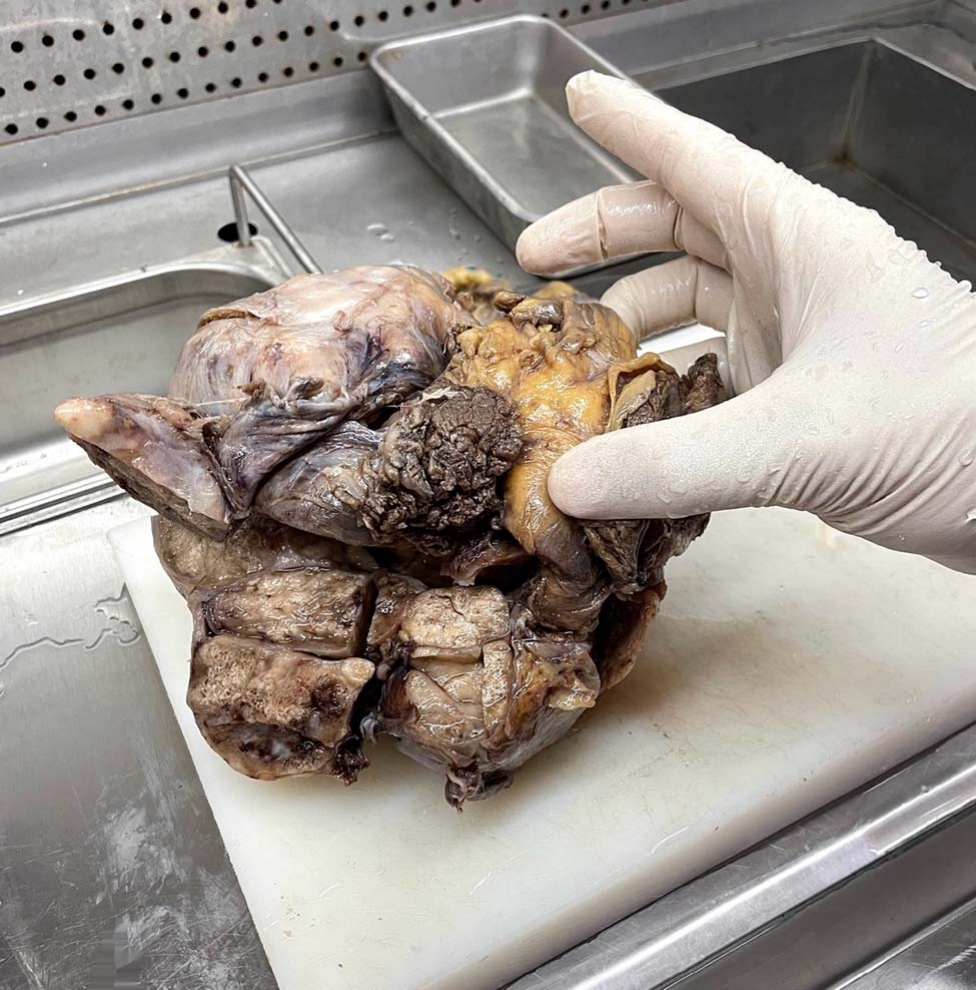
The tumor was 15×15×10 cm, with an attached bone (12th rib).

On macroscopic examination, the mass was oval in shape and well‑circumscribed, with an attached bone (12th rib) (Fig. 2). The tumor’s cut surface exhibited a multinodular/plexiform growth pattern with a firm consistency and creamy-heterogenous appearance.

**Figure 3:**
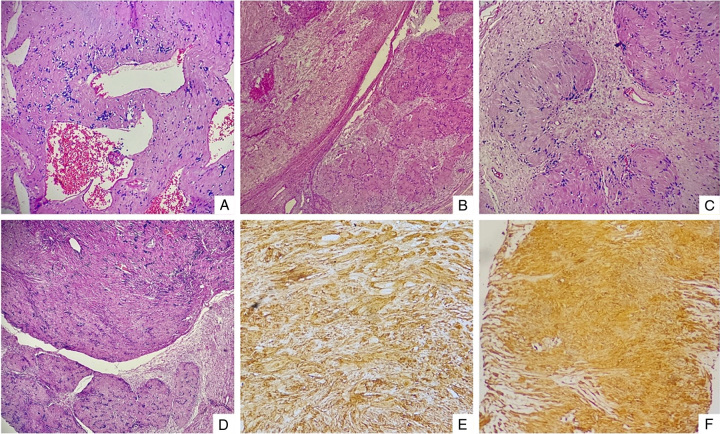
(A–D) Postoperative histopathology showing that the tumor is composed mainly of two patterns of hypercellular Antoni A and less cellular areas of Antoni B with inflammatory cells, delicate collagen bundles, foci of xanthomatous changes, and large irregularly spaced vessels, some with hyalinization and thrombosis. (E and F) Immunohistochemically, the tumor cells were strongly positive for S-100 protein.

The postoperative histopathological examination revealed a biphasic benign neural neoplasm with distinct patterns. It consisted of Antoni A regions, characterized by compact spindle cells exhibiting twisted nuclei and arranged in interlacing fascicles with nuclear palisading, known as Verocay bodies. Additionally, there were Antoni B areas, which were less cellular and featured inflammatory cells, delicate collagen bundles, patches of xanthomatous changes, and large irregularly spaced vessels, some exhibiting hyalinization and thrombosis. Although there were foci of nuclear atypia, no apparent mitosis or tumoral necrosis was observed. Immunohistochemistry confirmed the histologic diagnosis, revealing diffuse strong S-100 positivity (both nuclear and cytoplasmic staining) in the tumor cells (Fig. 3).

This comprehensive assessment led to the diagnosis of a giant ancient plexiform schwannoma. Notably, after a 2-year follow-up, the patient remained asymptomatic, and no signs of recurrence were observed.

## Discussion

RSs are unusual tumors, accounting for only 1–3% of all schwannomas^10^, yet they have the potential for considerable growth. The clinical presentation is unspecific, with masses less than 5 cm often discovered incidentally. Occasionally, they can lead to lower back pain and functional disturbances in adjacent gastrointestinal and urinary organs due to compression of the neighboring organs^7,11^.

Giant RSs are distinctive due to their rarity and the complexities involved in their management. Their presentation often includes various possible differential diagnoses, such as paraganglioma and pheochromocytoma, alongside conditions like fibrosarcoma, liposarcoma, and ganglioneuroma. The primary treatment encompasses complete surgical removal while preserving neurological function and minimizing postoperative complications. Preoperative evaluation is crucial, including lab and radiological exams. Complex tumor extensions require a multidisciplinary team of experts from diverse medical specialties, including surgeons, pathologists, radiologists, and oncologists, to collaborate and ensure effective case management and coordination^12,13^. Thus, efficient resource utilization is vital for optimizing surgical outcomes. Table 1 shows a summary of giant retroperitoneal schwannoma cases reported in the literature.

**Table 1:** Summary of giant retroperitoneal schwannoma cases reported.

References	Patient	Size	Location MRI/CT	Symptoms/Complaint
Algahtany *et al*. 2021^14^	A 56-year-old female	11×9×11 cm	A retroperitoneal mass that has severely damaged the L4 vertebra and caused neural compression	Left leg radicular pain, back pain, and loss of sensation along the L4 dermatome
Lamris *et al*. 2021^15^	A 25-year-old female	11×6.9 cm	Significant intra-abdominal mass in right para-umbilical region displaces adjacent ureter, contacting psoas and rectus abdominis muscles	Abdominal pain that was localized in the right flank
Gao *et al*. 2020^7^	A 69-year-old female	20×15×10 cm	A cystic mass encapsulated within the pelvis and hypogastric region	Recurring abdominal discomfort and constipation over a period of several months
Chen *et al*. 2015^16^	A 52-year-old female	10×9×8 cm	A heterogeneous mass with a well-defined border in the left lower quadrant	Palpable abdominal mass in left lower quadrant, present for six months, with progressive growth in 2 weeks
Fu *et al*. 2015^6^	A 71-year -old female	15×11×6 cm	Mass located between liver and upper pole of right kidney, potentially originating from liver, right adrenal gland, or right kidney	Asymptomatic , found increasing in size of tumor over 8 years incidentally
Khandakar *et al*. 2014^17^	A 65-year-old male	15×10×8 cm	A clearly defined heterogeneous solid mass in the retroperitoneal compressing the bladder and rectum	Altered bowel habit and abdominal discomfort for 6 months
Ozbir *et al.* 2011^18^	A 73-year-old male. A 46-year-old female	21×18×11 cm 17×15×11 cm	An enhancing solid mass encapsulated with areas of cystic and necrotic tissue, Originating from the retroperitoneum Solid mass from the lower porta hepatis extends to the iliac crest, compressing the right kidney between the superior and inferior vena cava	Abdominal mass, swelling, nonspecific abdominal pain 2-year history of flank pain, one episode of rectal hemorrhage, and progressive lower extremity edema

Schwannoma recurrences after resection are considered unfavorable. They often occur due to atypical histopathological evidence (increased mitotic activities or tumoral necrosis)^19,20^. However, Benign schwannomas can also potentially recur, with reported rates ranging from 5 to 10%. Achieving complete radical resection of RS is challenging due to the risk of bleeding, attributed to their highly vascularized nature, as well as the potential adhesion to retroperitoneal vessels^11,21^.

Schwannomas are solitary, encapsulated tumors with a firm texture, smooth surface, and gray color. Histologically, these tumors feature elongated spindle cells forming both hypercellular Antoni A areas characterized by twisted nuclei and Verocay bodies, as well as hypocellular Antoni B regions containing inflammatory cells, collagen bundles, xanthomatous changes, and irregular vessels. Ancient schwannomas, although rare, can exhibit degenerative changes like hemorrhage, cyst formation, and hyalinization. The key to their diagnosis is the prominent and widespread staining of S-100 protein in the tumor cells^18^. This unique subtype of schwannoma, known as ʻancient schwannomaʼ, was first described by Ackerman and Taylor. It is characterized by long-standing schwannomas with reduced Antoni A regions and increased hypocellular areas^22^.

Ancient schwannomas have been documented in the literature with various accompanying changes, including hyalinization, calcification, necrosis, hemorrhage, cystic formation, fatty degeneration, and infiltration by siderophages and histiocytes to varying extents. These alterations are believed to result from the tumor’s aging process. Occasionally, nuclear atypia and pleomorphism in these tumors can lead to the misdiagnosis of malignancy. In such cases, the low mitotic count is critical for accurate assessment^17^.

Preoperative identification of RS presents a significant challenge. Conventional imaging methods such as CT, MRI, and ultrasound lack specific features, making the diagnostic process intricate. Among them, abdominal MRI is the preferred choice due to its heightened diagnostic precision compared to ultrasound and CT scans. However, it is worth noting that accurately identifying RS preoperatively remains a challenge, with less than 20% of cases achieving definite diagnoses. Additionally, core needle biopsies may yield inconclusive or misleading results, especially for ancient and cystic schwannomas^11,13,14^.

In the patient’s case, the attempt at a definitive diagnosis through an ultrasound-guided biopsy proved unsuccessful due to insufficient tissue for subsequent immunohistochemical analysis. It is imperative to note that percutaneous biopsies carry inherent risks, including the potential for infection, hemorrhage, and the inadvertent seeding of tumor cells. Additionally, the presence of cellular pleomorphism in degenerated regions may sometimes lead to misinterpretation as malignancy. Hence, this study underscores the consensus that the ultimate diagnosis should primarily rely on the findings derived from the postoperative histopathological examination^16^.

## Conclusion

This unique case of a giant ancient plexiform retroperitoneal schwannoma highlights the complexity of this rare condition. A careful assessment for mitotic activity is necessary for this benign tumor variant. Our findings contribute to the existing knowledge regarding this distinct tumor entity’s successful treatment and clinical behavior. Further research and reporting will improve diagnostic capabilities and refine management strategies for ancient schwannoma patients.

## Ethics approval

The institutional board review at Guilan University of Medical Sciences granted approval for the study is available upon request from the corresponding author (Ethics approval code: IR.GUMS.REC.1402.263).

## Consent

Written informed consent was obtained from the patient to publish this case report and accompanying images. A copy of written consent is available for review by the Editor-in-Chief of this journal on request.

## Sources of funding

None.

## Author contribution

All authors contributed equally.

## Conflicts of interest disclosure

The authors declare that they have no competing interests.

## Research registration unique identifying number (UIN)

researchregistry9404.

https://www.researchregistry.com/browse-the-registry#home/.

## Guarantor

Elahe Abbaspour, Poursina Hospital, Rasht 41937 13194, Iran. E-mail: Elahe.abbaspourmd@gmail.com Orcid ID: 0000-0002-7387-8223.

## Data availability statement

The data used to support the findings of this case report are available from the corresponding author upon request. Anonymized and aggregated data that do not compromise patient confidentiality can be made available to researchers for further analysis upon request and appropriate ethical approvals.

## Provenance and peer review

Not invited.
